# Survival benefit of preoperative hepatic arterial infusion of oxaliplatin, fluorouracil, and leucovorin followed by hepatectomy for hepatocellular carcinoma

**DOI:** 10.3389/fphar.2023.1210835

**Published:** 2023-06-29

**Authors:** Zili Hu, Zhenyun Yang, Yangxun Pan, Yizhen Fu, Jiongliang Wang, Zhongguo Zhou, Minshan Chen, Dandan Hu, Yaojun Zhang

**Affiliations:** ^1^ Department of Liver Surgery, Sun Yat-Sen University Cancer Center, Guangzhou, China; ^2^ State Key Laboratory of Oncology in South China, Collaborative Innovation Center for Cancer Medicine, Sun Yat-Sen University Cancer Center, Guangzhou, China

**Keywords:** hepatic arterial infusion chemotherapy (HAIC), hepatocellular carcinoma (HCC), preoperative treatment, FOLFOX, nomogram

## Abstract

**Background:** Hepatic arterial infusion chemotherapy (HAIC) with cisplatin, fluorouracil, and leucovorin (FOLFOX) demonstrated promising efficacy against advanced hepatocellular carcinoma (HCC) as an alleviative treatment. We aimed to explore the survival benefit of preoperative FOLFOX-HAIC and establish a predictive nomogram.

**Methods:** This study retrospectively reviewed data from 1251 HCC patients who underwent liver resection. 1027 patients received liver resection alone (LR group), and 224 patients were treated with FOLFOX-HAIC followed by liver resection (HLR group). Propensity score matching (PSM) was conducted between the two groups. The nomogram was established based on the findings of the multivariable Cox regression analysis.

**Results:** After Propensity score matching according to initial tumor characteristics, the 1-, 2-, and 3-year overall survival rates were 85.4, 72.0, and 67.2% in the LR group and 95.2, 84.7, and 75.9% in the HLR group, respectively (*p* = 0.014). After PSM according to preoperative tumor characteristics, the 1-, 2-, and 3-year OS rates were 87.9, 76.6, and 72.3% in the LR group and 95.4, 84.4, and 75.1% in the HLR group, respectively (*p* = 0.24). Harrell’s C-indexes of the nomogram for OS prediction in patients with preoperative FOLFOX-HAIC were 0.82 (95% CI 0.78–0.86) in the training cohort and 0.87 (95% CI 0.83–0.93) in the validation cohort and the nomogram performed well-fitted calibration curves.

**Conclusion:** Preoperative FOLFOX-HAIC is associated with a longer survival outcome for HCC patients. The novel nomogram efficiently predicted the OS of patients who underwent preoperative FOLFOX-HAIC.

## 1 Introduction

Hepatocellular carcinoma (HCC) ranks seventh among the most prevalent cancer types and is the third most common cause of cancer-related mortality worldwide (Sung et al, 2021). Liver resection is considered to be one of the curative therapies of HCC (Arii et al, 2000; Forner et al, 2018; Benson et al, 2021a). However, less than 30% of patients with HCC are surgical candidates at the initial diagnosis in China (Park et al, 2015).

Hepatic arterial infusion chemotherapy (HAIC) is recommended as one of the alleviative treatments for advanced HCC (Chen et al, 2020; Kudo et al, 2021). However, the optimal regimen for HAIC remains controversial. HAIC with cisplatin, fluorouracil, and leucovorin (FOLFOX) has been widely used in Asia (Tsai et al, 2014). Recently, FOLFOX-HAIC has demonstrated promising therapeutic effects for advanced HCC in several studies (He et al, 2017; Lyu et al, 2018; He et al, 2019; Li et al, 2022; Lyu et al, 2022), including prospective randomized trials (Li et al, 2022; Lyu et al, 2022). Li et al (2022)’s study demonstrated that FOLFOX-HAIC elicited a higher objective response rate and fewer adverse events than transhepatic arterial chemotherapy and embolization (TACE) for large HCC. Lyu et al (2022)’s study showed that FOLFOX-HAIC provided a higher efficacy and better survival outcome than sorafenib in locally advanced HCC. In addition, FOLFOX-HAIC led to a better chance of downstaging in advanced HCC cases (Li et al, 2022; Lyu et al, 2022). FOLFOX-HAIC is regarded as a conversion therapy before surgical resection to exert a significant effect in reducing the tumor size.

Patients suffering from several malignancies including lung cancer (Ettinger et al, 2022), colorectal cancer (Benson et al, 2021b) and breast cancer (Gradishar et al, 2020) are recommended to receive preoperative treatments like chemotherapy or radiotherapy, to improve the therapeutic effect of surgery. However, efficient preoperative treatments for HCC are still lacking. In this study, we aimed to explore whether preoperative FOXFOL-HAIC improves outcomes for HCC patients in comparison to those who underwent liver resection alone. In addition, we developed a nomogram to predict the overall survival (OS) of patients who underwent FOXFOX-HAIC followed by liver resection. The novel nomogram can guide surgeons to select the proper candidates to receive liver resection after FOXFOL-HAIC.

## 2 Methods

### 2.1 Patients

This study retrospectively reviewed data from consecutive HCC patients who underwent liver resection at the Sun Yat-Sen University Cancer Center (SYSUCC) between January 2017 and January 2021. The diagnosis of HCC was according to the imaging studies (contrast enhanced computed tomography and/or magnetic resonance imaging) presenting both early enhancement and delayed decreased enhancement, in compliance with the American Association for the Study of Liver Diseases Practice Guideline for Management of HCC (Bruix et al, 2011). The inclusion criteria were as follows: 1) Age 18–75 years; 2) No history of other malignancies; 3) Child-Pugh score of 5–7; 4) Performance status of 0 or 1; 5) Absence of extrahepatic metastasis; and 6) Initial diagnosis of HCC. Patients with a previous history of liver resection, ablation, TACE, immune checkpoint inhibitors, or targeted therapy were excluded. Finally, 1,251 patients were included, of which 1,027 patients received liver resection alone (LR group), and 224 patients were treated with FOLFOX-HAIC followed by liver resection (HLR group). This study was conducted according to the ethical guidelines of the 1975 Declaration of Helsinki and was approved by the institutional review board of SYSUCC.

### 2.2 HAIC procedures

HAIC was administered in 3-week cycles. On day 1 of each cycle, femoral artery puncture and catheterization were performed, followed by infusion of the following regimen into the hepatic artery: oxaliplatin (130 mg/m^2^) from hours 0–2 on day 1, leucovorin (400 mg/m^2^) from hours 2-3 on day 1, and fluorouracil (400 mg/m^2^ bolus at hour 3 on day 1 and 2,400 mg/m^2^ over 24 h). The catheter and sheath were removed immediately following completion of each HAIC cycle, and repetitive catheterization was performed in subsequent cycles. To evaluate treatment efficacy, magnetic resonance imaging (MRI) was conducted every 6 weeks during the preoperative HAIC period, and efficacy was assessed according to the modified Response Evaluation Criteria in Solid Tumors (mRECIST) guidelines (Lencioni and Llovet, 2010). Following estimation of the treatment response, patients were evaluated for curative resection to determine if removal of all tumors was feasible while ensuring sufficient hepatic functional reserve. In cases where patients were assessed as having progressive disease (PD), hepatectomy was still considered if curative resection was achievable.

### 2.3 Follow-up

Following surgery, all patients underwent follow-up evaluations at 1 month, and then at 3-month intervals for 2 years. Thereafter, follow-up evaluations were conducted every 6 months. These evaluations included laboratory tests, such as serum alpha-fetoprotein (AFP) level, liver function tests, and blood tests, as well as MRI scans. In case of recurrence, treatment for recurrent HCC was determined based on the patient’s liver function and the status of the tumor recurrence. Treatment options included further surgical resection, radiofrequency ablation, interventional therapy, or targeted drug therapy.

### 2.4 Outcomes and definitions

The primary endpoint was OS, which was defined as the time interval between liver resection and either death from any cause or the last follow-up date. The secondary endpoint was recurrence-free survival (RFS), which was defined as the duration between the date of liver resection and the date of HCC recurrence. Preoperative HAIC was defined as patients who completed HAIC treatment before their clinical admission for liver resection. The extent of surgical resection was defined according to Couinaud’s classification system. The histologic grade of tumor differentiation was categorized based on Edmondson–Steiner (ES) classification (Edmondson and Steiner, 1954). The definition of the overall response rate (ORR) is the combined rate of complete response (CR) and partial response (PR). Hepatitis B virus (HBV) infection was defined as the presence of hepatitis B surface antigen (HBsAg) for more than 6 months prior to the diagnosis of HCC. Cirrhosis was histologically diagnosed based on the liver specimens obtained during resection.

### 2.5 Statistical analysis

Statistical analyses were conducted using R version 3.6.3 (R Foundation for Statistical Computing, Vienna, Austria, https://www.R-project.org/) and SAS version 26.0 (SAS Institute, Cary, NC). Categorical variables were presented as frequencies and percentages, and the chi-square test was used to compare differences between the two groups. Continuous variables were described as either mean ± standard deviation or median with interquartile range for parametric and nonparametric variables, respectively. Student’s t-test or nonparametric tests were used to compare continuous variables. Survival curves were generated using the Kaplan-Meier method and compared using the log-rank test. Propensity score matching (PSM) was conducted using the “MatchIt” R package, with a caliper width set to 0.2 of the standard deviation of the logit of the propensity score. Univariate and multivariate Cox proportional hazards models were used to evaluate risk factors for both recurrence and OS. A nomogram was developed based on the risk factors identified through multivariate analysis and generated using the “rms” R package. The calibration curve was generated using regression analysis.

## 3 Results

### 3.1 Baseline characteristics of the patients

This study consecutively collected 1,251 patients with a median age of 53.7 [95% confidence interval (CI), 41.8–65.6] y. 1,076 (86.0%) patients were male. The average tumor size was 6.15 (95% CI, 2.48–9.82) cm. A total of 1,042 (83.3%) patients were infected with HBV and 778 (62.2%) were confirmed to have cirrhosis.

Compared to the LR group, the HLR group contained significantly more patients with larger tumor sizes (9.0 vs. 5.5 cm, *p* < 0.001), longer prothrombin time (PT, 12.0 vs. 11.8 s, *p* = 0.008), higher levels of alanine aminotransferase (ALT, 53.8 vs. 42.9 U/L, *p* = 0.023), aspartate aminotransferase (AST, 59.2 vs. 40.0 U/L, *p* < 0.001), higher AFP positivity ratio (59.8% vs. 34.2%), more patients with HBV infection (90.2% vs. 84.4%, *p* = 0.027), more patients with multiple tumors (35.3% vs. 21.6%, *p* < 0.001), more patients with albumin-bilirubin (ALBI) grade 2 (14.3% vs. 8.4%, *p* = 0.006) and more patients with portal vein tumor thrombus (PVTT, 28.6% vs. 6.7%, *p* < 0.001) (Table 1). PSM (1:1 matching) according to initial tumor size, initial tumor number, ALBI grade, and PVTT analysis generated a cohort (initial PSM cohort) of 224 and 224 patients in the LR and the HLR groups, respectively. The characteristics of the two groups were balanced, with a standardized mean difference of less than 10% for all baseline variables except microvascular invasion and cirrhosis (Table 2). Similarly, PSM (1:1 matching) according to preoperative tumor size, preoperative tumor size, ALBI grade, and PVTT analysis generated a new cohort (preoperative PSM cohort) of 214 and 214 patients in the LR and the HLR groups, respectively. Although the preoperative levels of albumin, total bilirubin, and ALT were lower in the HLR group, no significant difference in ALBI grade was observed between the two groups (Table 3).

**TABLE 1:** Baseline characteristics of all patients (*n* = 1,251).

	LR group	HLR group	*p*-value
	(*n* = 1,027)	(*n* = 224)	
Age (years)	54.1 ± 11.8	51.8 ± 12.3	0.012
Gender (N, %)			
women	141 (13.7)	34 (15.2)	0.571
men	886 (86.3)	190 (84.8)	
HBV infection (N, %)			0.027
absence	155 (15.6)	22 (9.8)	
presence	840 (84.4)	202 (90.2)	
MVI (N, %)			<0.001
absence	637 (62.0)	180 (80.4)	
presence	390 (38.0)	44 (19.6)	
Cirrhosis (N, %)			<0.001
absence	359 (35)	114 (50.9)	
presence	668 (65)	110 (49.1)	
Tumor size (cm)	5.5 ± 3.5	9.0 ± 3.3	<0.001
Tumor number (N, %)			<0.001
solitary	805 (78.4)	145 (64.7)	
multiple	222 (21.6)	79 (35.3)	
Differentiation (N, %)			0.278
I, II	569 (55.4)	133 (59.4)	
III, IV	458 (44.6)	91 (40.6)	
Platelet (N, %)			<0.001
>100 × 10^3^/mm^3^	968 (94.3)	224 (100.0)	
≤100 × 10^3^/mm^3^	59 (5.7)	0 (0.0)	
PT(s)	11.8 ± 0.9	12.0 ± 0.9	0.008
Albumin (g/dL)	44.1 ± 12.9	43.0 ± 3.8	0.217
Total bilirubin (mg/dL)	13.2 ± 5.1	14.1 ± 6.0	0.024
ALT (U/L)	42.9 ± 70.0	53.8 ± 39.7	0.023
AST (U/L)	40.0 ± 41.8	59.2 ± 40.5	<0.001
AFP (N, %)			<0.001
<400 ng/mL	676 (65.8)	90 (40.2)	
≥400 ng/mL	351 (34.2)	134 (59.8)	
ALBI (N, %)			0.006
Grade 1	941 (91.6)	192 (85.7)	
Grade 2	86 (8.4)	32 (14.3)	
PVTT			<0.001
absence	958 (93.3)	160 (71.4)	
presence	69 (6.7)	64 (28.6)	

Categorical variables are described as frequencies and percentages. Continuous variables are described as mean ± standard deviation and median with interquartile range for parametric and non-parametric variables, respectively. AFP, alpha fetoprotein; ALBI, Albumin-Bilirubin; ALT, alanine aminotransferase; AST, aspartate aminotransferase; HBV, hepatitis B virus; MVI: microvascular invasion, PSM: propensity score matching; PT, prothrombin time; PVTT, portal vein tumor thrombus.

**TABLE 2:** Baseline characteristics of patients in the initial PSM cohort (*n* = 448).

	LR group	HLR group	*p*-value
	(*n* = 224)	(*n* = 224)	
Age (years)	52.9 ± 11.6	51.8 ± 12.3	0.311
Gender (N, %)			0.412
women	28 (12.5)	34 (15.2)	
men	196 (87.5)	190 (84.8)	
HBV infection (N, %)			0.113
absence	33 (15.1)	22 (9.8)	
presence	191 (85.3)	202 (90.2)	
MVI (N, %)			<0.001
absence	84 (37.5)	181 (80.8)	
presence	140 (62.5)	43 (19.2)	
Cirrhosis (N, %)			<0.001
absence	78 (34.8)	114 (50.9)	
presence	146 (65.2)	110 (49.1)	
Tumor size (cm)	8.8 ± 3.8	9.0 ± 3.3	0.640
Tumor number (N, %)			0.379
solitary	136 (60.7)	145 (64.7)	
multiple	88 (39.3)	79 (35.3)	
Differentiation (N, %)			0.566
I, II	127 (56.7)	133 (59.4)	
III, IV	97 (43.3)	91 (40.6)	
Platelet (N, %)			0.002
>100 × 10^3^/mm^3^	215 (96.0)	224 (100.0)	
≤100 × 10^3^/mm^3^	9 (4.0)	0 (0.0)	
PT(s)	12.1 ± 0.9	12.0 ± 0.9	0.331
Albumin (g/dL)	42.6 ± 3.7	43.0 ± 3.8	0.259
Total bilirubin (mg/dL)	13.3 ± 5.0	14.1 ± 6.0	0.113
ALT (U/L)	45.1 ± 47.6	53.8 ± 39.7	0.38
AST (U/L)	52.7 ± 48.1	59.2 ± 40.5	0.124
AFP (N, %)			0.87
<400 ng/mL	108 (48.2)	90 (40.2)	
≥400 ng/mL	116 (51.8)	134 (59.8)	
ALBI (N, %)			0.598
Grade 1	188 (83.9)	192 (85.7)	
Grade 2	36 (16.1)	32 (14.3)	
PVTT			0.456
absence	167 (74.6)	160 (71.4)	
presence	57 (25.4)	64 (28.6)	

Categorical variables are described as frequencies and percentages. Continuous variables are described as mean ± standard deviation and median with interquartile range for parametric and non-parametric variables, respectively. AFP, alpha fetoprotein; ALBI, Albumin-Bilirubin; ALT, alanine aminotransferase; AST, aspartate aminotransferase; HBV, hepatitis B virus; MVI, microvascular invasion; PSM, propensity score matching; PT, prothrombin time; PVTT, portal vein tumor thrombus.

**TABLE 3:** Baseline characteristics of patients in the preoperative PSM cohort (*n* = 428).

	LR group	HLR group	*p*-value
	(*n* = 214)	(*n* = 214)	
Age (years)	55.5 ± 11.6	51.9 ± 12.2	0.002
Gender (N, %)			0.158
women	24 (11.2)	34 (15.9)	
men	190 (88.8)	180 (84.1)	
HBV infection (N, %)			0.025
absence	37 (17.9)	22 (10.3)	
presence	170 (82.1)	192 (89.7)	
MVI (N, %)			<0.001
absence	105 (49.1)	171 (80.7)	
presence	109 (50.9)	43 (19.3)	
Cirrhosis (N, %)			<0.001
absence	60 (28.0)	110 (51.6)	
presence	154 (69.2)	104 (48.4)	
Tumor size (cm)	7.2 ± 3.8	6.6 ± 2.9	0.108
Tumor number (N, %)			0.758
solitary	142 (66.4)	145 (67.8)	
multiple	72 (33.6)	69 (32.2)	
Differentiation (N, %)			0.943
I, II	120 (56.1)	127 (59.3)	
III, IV	94 (43.9)	87 (40.7)	
Platelet (N, %)			0.049
>100 × 10^3^/mm^3^	208 (97.2)	198 (92.5)	
≤100 × 10^3^/mm^3^	6 (2.8)	16 (7.5)	
PT(s)	12.0 ± 0.9	12.1 ± 0.9	0.826
Albumin (g/dL)	42.8 ± 3.5	41.3 ± 3.4	<0.001
Total bilirubin (μmol/L)	13.2 ± 5.0	11.3 ± 5.0	<0.001
ALT (U/L)	44.7 ± 55.7	33.4 ± 27.0	0.001
AST (U/L)	46.1 ± 55.7	42.6 ± 26.5	0.287
AFP (N, %)			0.618
<400 ng/mL	136 (63.6)	131 (61.2)	
≥400 ng/mL	78 (36.4)	83 (38.8)	
ALBI (N, %)			0.358
Grade 1	183 (85.5)	176 (82.2)	
Grade 2	31 (14.5)	38 (17.8)	
PVTT			0.99
absence	159 (74.3)	159 (71.4)	
presence	55 (25.7)	55 (28.6)	

Categorical variables are described as frequencies and percentages. Continuous variables are described as mean ± standard deviation and median with interquartile range for parametric and non-parametric variables, respectively. AFP, alpha fetoprotein; ALBI, Albumin-Bilirubin; ALT, alanine aminotransferase; AST, aspartate aminotransferase; HBV, hepatitis B virus; MVI, microvascular invasion, PSM, propensity score matching; PT, prothrombin time; PVTT, portal vein tumor thrombus.

### 3.2 Survival benefit of preoperative FOLFOX-HAIC followed by hepatectomy

**FIGURE 1:**
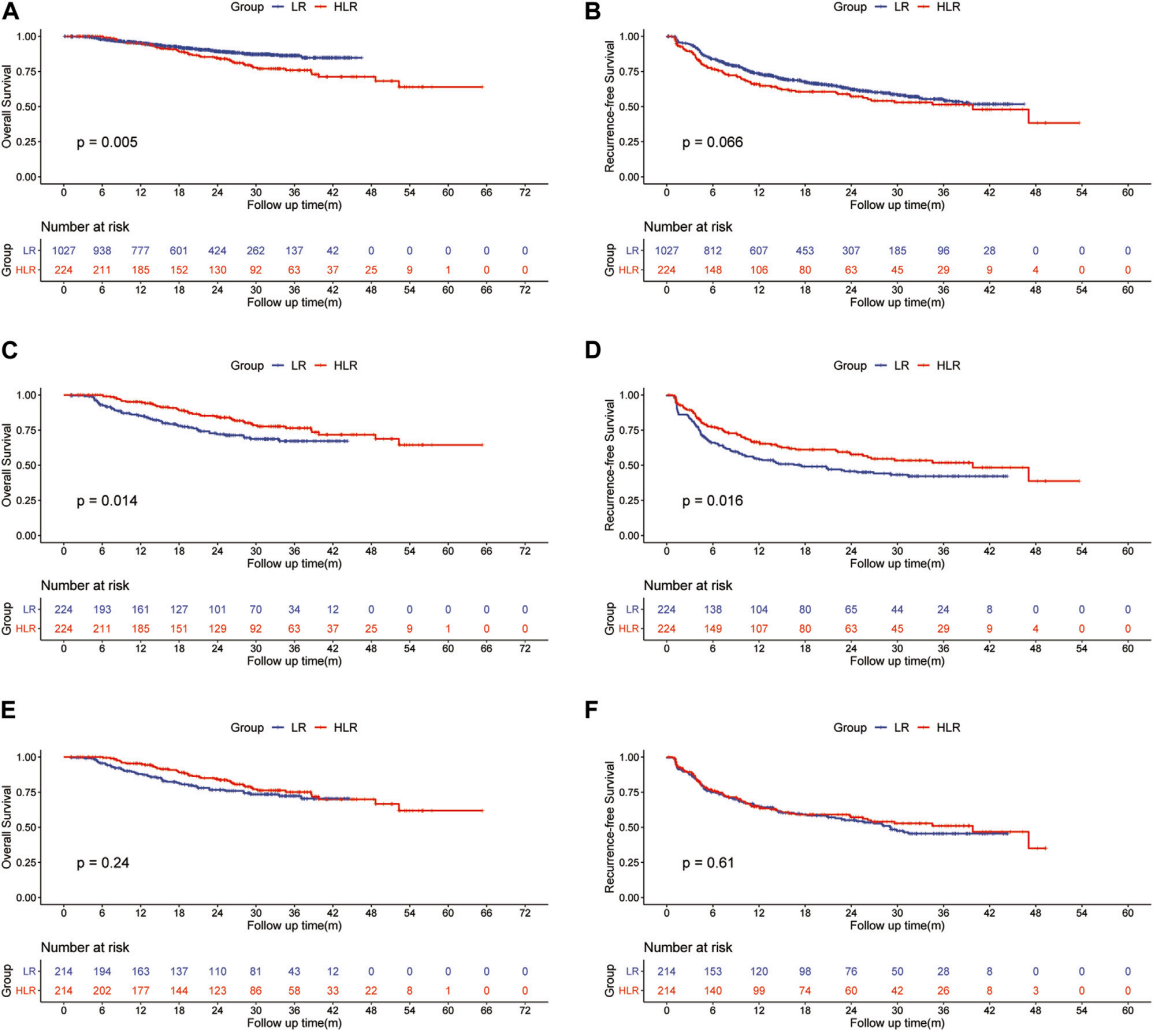
Kaplan—Meier curves of HCC overall survival **(A)** and recurrence **(B)** between the LR group and the HLR group in all patients; Kaplan—Meier curves of HCC overall survival **(C)** and recurrence **(D)** between the LR group and the HLR group in the initial PSM cohort; Kaplan—Meier curves of HCC overall survival **(E)** and recurrence **(F)** between the LR group and the HLR group in the preoperative PSM cohort.

For patients in the entire cohort, the 1-, 2-, and 3-year OS rates were 95.1, 89.2, and 86.3% in the LR group and 95.2%, 84.7, and 75.9% in the HLR group, respectively (Figure 1A). Patients in the HLR group demonstrated worse survival compared to those in the LR group (*p* = 0.005) probably owing to more patients with larger tumors, multiple tumors, ALBI grade 2, and PVTT (*p* < 0.001, *p* < 0.001, *p* = 0.006, and *p* < 0.001, respectively) in the HLR group. The 1-, 2-, and 3-year RFS rates of HCC were 73.4, 62.2, and 54.8% in the LR group and 66.0, 57.1, and 51.4% in the HLR group, respectively (Figure 1B). No significant difference between the two groups in RFS (*p* = 0.066) was observed.

For patients in the initial PSM cohort, the 1-, 2-, and 3-year OS rates were 85.4, 72.0, and 67.2% in the LR group and 95.2, 84.7, and 75.9% in the HLR group, respectively (Figure 1C). OS was significantly higher in the HLR group than that in the LR group (*p* = 0.014). A multivariate Cox regression analysis was performed on the initial PSM cohorts, and consequently, preoperative FOLFOX-HAIC was identified as a significant protective factor for survival (HR 0.525; 95% CI 0.343–0.803; *p* = 0.003). In addition, tumor size, tumor number, microvascular invasion (MVI), PT, and PVTT were identified as significant factors associated with survival (Table 4). The 1-, 2-, and 3-year RFS rates of HCC were 54.1, 45.7, and 42.1% in the LR group and 66.0, 57.1, and 51.4% in the HLR group, respectively (Figure 1D). The HLR group exhibited significantly lower recurrence rates than the LR group (*p* = 0.016). A multivariate Cox regression analysis was performed on the initial PSM cohorts and preoperative FOLFOX-HAIC was identified as a significant protective factor for recurrence (HR 0.681; 95% CI 0.508–0.914; *p* = 0.01). In addition, tumor size, tumor number, tumor differentiation, MVI, and PVTT were identified as crucial factors associated with recurrence (Table 4).

**TABLE 4:** Univariate and multivariate analysis of risk factors for overall survival and recurrence-free survival in the initial PSM cohort.

Variables	Overall survival								Recurrence-free survival							
	Univariate analysis			Multivariate analysis					Univariate analysis					Multivariate analysis		
	HR	95% CI	*p*	HR		95% CI		*p*-Value	HR		95% CI	*p*		HR	95% CI	*p*
Age (years)	0.977	(0.961–0.993)	0.004	0.988		(0.970–1.006)		0.179	0.980		(0.97–0.991)	<0.001		0.952	(0.688–1.317)	0.768
Sex (female: male)	0.960	(0.545–1.691)	0.888						0.709		(0.527–1.221)	0.303				
Tumor size (cm)	1.126	(1.068–1.186)	<0.001	1.109		(1.021–1.171)		<0.001	0.107		(1.03–1.113)	<0.001		1.049	(1.006–1.093)	0.024
Tumor number (solitary: multiple)	1.584	(1.068–2.349)	0.022	1.619		(1.091–2.404)		0.017	1.630		(1.237–2.149)	<0.001		1.735	(1.312–2.293)	<0.001
Tumor differentiation (I II: III IV)	2.022	(1.35–3.031)	<0.001	1.505		(0.981–2.311)		0.061	1.775		(1.342–2.347)	<0.001		1.520	(1.131–2.043)	0.006
Cirrhosis (no: yes)	0.845	(0.571–1.251)	0.401						0.998		(0.755–1.318)	0.769				
MVI (no: yes)	2.739	(1.875–4.001)	<0.001	2.406		(1.594–3.631)		<0.001	2.367		(1.822–3.074)	<0.001		2.240	(1.689–2.971)	<0.001
HBV infection (no: yes)	1.683	(0.816–3.468)	0.158						1.065		(0.700–1.619)	0.769				
Platelet, ×109/L (≥100:<100) (≥100:<100)	2.446	(0.341–17.556)	0.374						0.954		(0.393–2.319)	0.918				
AFP, ng/mL (<400: ≥400)	1.736	(1.143–2.635)	0.01	1.499		(0.977–2.300)		0.064	1.380		(1.041–1.830)	0.025		1.228	(0.918–1.641)	0.166
ALT, U/L (≤50:>50)	0.996	(0.654–1.516)	0.984						0.982		(0.729–1.324)	0.906				
AST, U/L (≤40:>40)	1.923	(1.259–2.939)	0.002	1.403		(0.888–2.217)		0.147	1.578		(1.186–2.101)	0.002		1.270	(0.929–1.737)	0.134
Albumin, g/L (≥35:<35)	1.010	(0.955–1.069)	0.717						1.006		(0.970–1.044)	0.747				
Total bilirubin, μmol/L (≤17.1:>17.1)	1.005	(0.972–1.04)	0.759						1.018		(0.995–1.042)	0.747				
PT, s (≤13.5; >13.5)	1.366	(1.112–1.679)	0.003	1.345		(1.094–1.652)		0.005	1.144		(0.986–1.328)	0.075				
ALBI grade (I: II)	1.609	(0.617–1.851)	0.812						1.065		(0.730–1.553)	0.746				
PVTT (no; yes)	1.716	(1.137–2.591)	0.01	1.647		(1.074–2.517)		0.022	2.367		(1.822–3.074)	<0.001		1.546	(1.139–2.099)	0.005
Neoadjuvant HAIC (no: yes)	0.606	(0.405–0.906)	0.015	0.525		(0.343–0.803)		0.003	0.709		(0.536–0.937)	0.016		0.681	(0.508–0.914)	0.01

AFP, alpha fetoprotein; ALBI, Albumin-Bilirubin; ALT, alanine aminotransferase; AST, aspartate aminotransferase; HAIC, hepatic arterial infusion chemotherapy; HBV, hepatitis B virus; MVI, microvascular invasion, PSM, propensity score matching; PT, prothrombin time; PVTT, portal vein tumor thrombus.

For patients in the preoperative PSM cohort, the 1-, 2-, and 3-year OS rates were 87.9, 76.6, and 72.3% in the LR group and 95.4, 84.4, and 75.1% in the HLR group, respectively (Figure 1E). The 1-, 2-, and 3-year RFS rates of HCC were 64.7, 55.1, and 45.5% in the LR group and 64.8, 57.2, and 51.0% in the HLR group, respectively (Figure 1F). Thus, no significant difference was observed in OS (*p* = 0.24) and RFS (*p* = 0.31) between the two groups.

Compared with the LR group, the HLR group had more patients with shorter postoperative hospital stays (9.3 vs. 11.6 days, *p* < 0.001; 9.2 vs. 11.5 days, *p* < 0.001) but longer operation time (178.3 vs. 156.7 min, *p* < 0.001; 177.6 vs. 158.0 min, *p* < 0.001) and hepatic portal occlusion time (25.4 vs. 11.6, *p* < 0.001; 25.4 vs. 11.6 min, *p* < 0.001) in both initial and preoperative PSM cohorts. Operative blood loss, postoperative complications, and 90-day mortality were not significantly different between the two groups in both initial and preoperative PSM cohorts. However, within the complications, the HLR group had more patients with hepatic insufficiency and bile leakage. Details are shown in Table 5.

**TABLE 5:** Surgical features and short-term outcome between the two groups.

	Initial PSM cohort			Preoperative PSM cohort		
	LR group	HLR group	*p*-value	LR group	HLR group	*p*-value
	(*n* = 224)	(*n* = 224)		(*n* = 214)	(*n* = 214)	
Postoperative hospital stays (days)	11.6 (8.9–14.3)	9.3 (5.8–12.8)	<0.001	11.5 (8.5–14.5)	9.2 (6.0–12.4)	<0.001
Operation time (min)	156.7 (110.5–202.9)	178.3 (129.4–227.2)	<0.001	158.0 (102.7–213.3)	177.6 (128.2–227)	<0.001
Operative blood loss (ml)	423.9 (7.7–840.1)	399.6 (27.9–771.3)	0.515	414.3 (−136.4–965)	398.8 (25.7–771.9)	0.735
Hepatic portal occlusion time (min)	11.6 (−2.1–25.3)	25.4 (13.3–37.5)	<0.001	11.6 (−1.7–24.9)	25.4 (13.2–37.6)	<0.001
Postoperative complications (N, %)			0.230			0.560
absent	203 (90.6)	195 (87.1)		189 (88.3)	185 (86.4)	
hepatic insufficiency	3 (1.3)	15 (6.7)		2 (0.9)	15 (7.0)	
bile leakage	1 (0.4)	7 (3.1)		1 (0.5)	7 (3.3)	
thorax/peritoneal effusion	10 (4.5)	7 (3.1)		9 (4.2)	7 (3.3)	
pulmonary/peritoneal infection	4 (1.8)	3 (1.3)		5 (2.3)	3 (1.4)	
postoperative hemorrhage	0 (0)	3 (1.3)		0 (0)	3 (1.4)	
intestinal obstruction	5 (2.2)	0 (0)		7 (3.3)	0 (0)	
others	2 (0.9)	1 (0.4)		4 (1.9)	1 (0.5)	
90-day mortality (N, %)	5 (2.2)	7 (3.1)	0.558	4 (1.9)	7 (3.3)	0.359

Categorical variables are described as frequencies and percentages. Continuous variables are described as mean ± standard deviation and median with interquartile range for parametric and non-parametric variables, respectively. Others of postoperative complications include incision dehiscence, lymphorrhagia, pneumothorax et al.

### 3.3 Prognostic factors associated with OS and RFS in patients treated with preoperative FOLFOX-HAIC

**FIGURE 2:**
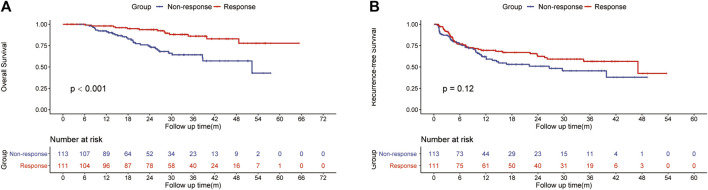
Kaplan—Meier curves of HCC recurrence **(A)** and overall survival **(B)** between the Response and the Non-response group.

**FIGURE 3:**
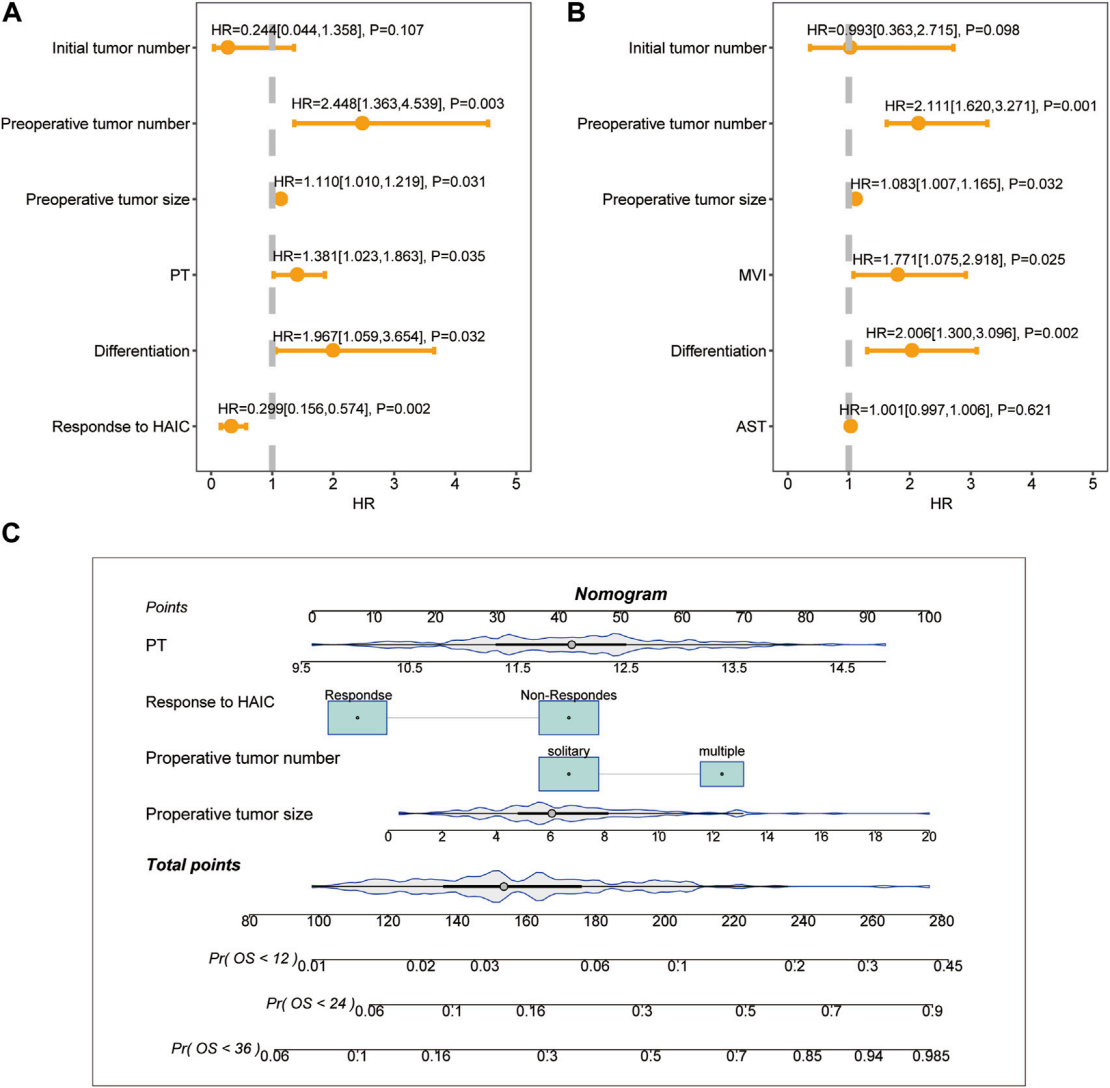
Multivariate analysis of risk factors for overall survival **(A)** and recurrence-free survival **(B)** in patients with preoperative FOLFOX-HAIC. Nomograms for predicting the 1-, 2- and 3-year mortality **(C)** rates in patients with preoperative FOLFOX-HAIC.

The ORR of FOLFOX-HIAC was 49.6%, 13 (5.8%) patients had CR, 98 (43.8%) had PR, 104 (46.4%) had stable disease (SD), and 9 (4.0%) had PD, as estimated according to Mrecist (Lencioni and Llovet, 2010). For patients in the HLR group, the 1-, 2-, and 3-year OS rates were 92.2, 74.5, and 64.2% in the Non-response group and 98.1, 93.7, and 86.1% in the Response group, respectively (Figure 2A). Thus, OS was notably higher in the Response group than in the Non-response group (*p* < 0.001). The 1-, 2-, and 3-year RFS rates of HCC were 62.1, 50.9, and 45.5% in the Non-response group and 69.5, 62.5, and 56.6% in the Response group, respectively (Figure 2B). No significant difference was observed in RFS between the two groups (*p* = 0.12). The multivariate Cox regression analysis was performed on the HLR group and the results indicated that preoperative tumor size, preoperative tumor number, tumor differentiation, PT, and response to HAIC were significant factors associated with OS (Figure 3A). The results also implied that preoperative tumor size, preoperative tumor number, tumor differentiation, and MVI were associated with RFS (Figure 3B) in patients treated with preoperative HAIC (Table 6).

**TABLE 6:** Univariate and multivariate analysis of risk factors for overall survival and recurrence-free survival in patients with preoperative FOLFOX-HAIC.

Variables	Overall survival								Recurrence-free survival							
	Univariate analysis			Multivariate analysis					Univariate analysis					Multivariate analysis		
	HR	95% CI	*p*	HR		95% CI		*p*-Value	HR		95% CI	*p*		HR	95% CI	*p*
Age (years)	0.981	(0.958–1.005)	0.117						0.990		(0.973–1.006)	0.222				
Sex (female: male)	0.771	(0.343–1.734)	0.529						0.999		(0.57–1.751)	0.997				
Initial tumor size (cm)	1.072	(0.982–1.171)	0.122						1.037		(0.973–1.106)	0.263				
Preoperative tumor size (cm)	1.218	(1.118–1.327)	<0.001	1.110		(1.010–1.219)		0.031	1.116		(1.044–1.194)	0.001		1.083	(1.007–1.165)	0.032
Initial tumor number (solitary: multiple)	1.82	(1.002–3.305)	0.049	0.244		(0.044–1.358)		0.107	1.693		(1.105–2.595)	0.016		0.993	(0.363–2.715)	0.989
Preoperative tumor number (solitary: multiple)	2.343	(1.293–4.245)	0.005	2.448		(1.363–4.539)		0.003	2.002		(1.306–3.07)	0.001		2.111	(1.62–3.271)	0.001
Tumor differentiation (I II: III IV)	1.845	(1.014–3.355)	0.045	1.967		(1.059–3.654)		0.032	2.125		(1.385–3.26)	<0.001		2.006	(1.300–3.096)	0.002
Cirrhosis (no: yes)	0.749	(0.412–1.362)	0.343						0.975		(0.64–1.484)	0.905				
MVI (no: yes)	1.714	(0.929–3.161)	0.085						2.100		(1.408–3.133)	<0.001		1.771	(1.075–2.918)	0.025
HBV infection (no: yes)	1.398	(0.432–4.527)	0.576						0.957		(0.392–1.468)	0.412				
Platelet, ×109/L (≥100:<100) (≥100:<100)	1.002	(0.999–1.005)	0.178						1.001		(0.999–1.004)	0.272				
AFP, ng/mL (<400: ≥400)	0.852	(0.468–1.551)	0.852						1.029		0.671–1.578)	0.896				
ALT, U/L (≤50:>50)	1.002	(0.995–1.009)	0.536						0.998		(0.992–1.004)	0.467				
AST, U/L (≤40:>40)	1.004	(0.998–1.010)	0.234						1.005		(1.001–1.008)	0.021		1.001	(0.997–1.006)	0.621
ALB, g/L (≥35:<35)	1.027	(0.94–1.123)	0.550						1.039		(0.979–1.102)	0.205				
TBIL, μmol/L (≤17.1:>17.1)	0.994	(0.947–1.043)	0.794						1.027		(0.996–1.059)	0.090				
PT, s (≤13.5; >13.5)	1.367	(1.009–1.85)	0.043	1.381		(1.023–1.863)		0.035	1.124		(0.902–1.401)	0.297				
ALBI grade (I: II)	0.561	(0.201–1.571)	0.271						0.632		(0.317–1.261)	0.193				
PVTT (no; yes)	1.576	(0.777–3.198)	0.208						1.527		(0.942–2.477)	0.086				
Respond to HAIC (no; yes)	0.299	(0.156–0.574)	<0.001	0.321		(0.154–0.670)		0.002	0.715		(0.467–1.093)	0.121				

AFP, alpha fetoprotein; CI, confidence interval; ALBI, Albumin-Bilirubin; ALT, alanine aminotransferase; AST, aspartate aminotransferase; HAIC, hepatic arterial infusion chemotherapy; HBV, hepatitis B virus; HR, hazard rate; MVI, microvascular invasion, PSM, propensity score matching; PT, prothrombin time; PVTT, portal vein tumor thrombus.

### 3.4 Development nomogram of OS in the HLR group

**FIGURE 4:**
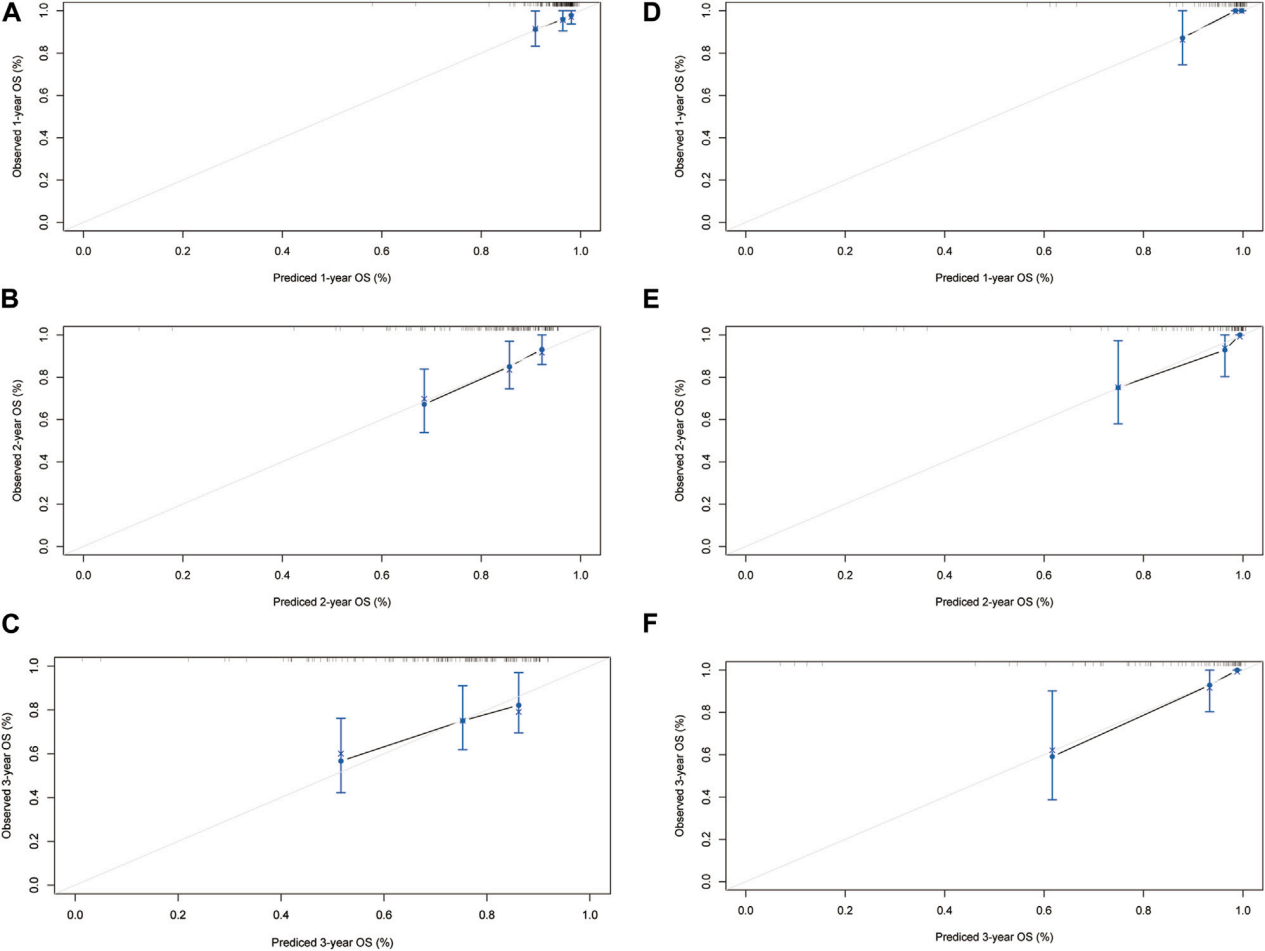
The calibration curves for predicting the 1-, 2- and 3-year mortality in the training cohort **(A–C)** and the internal validation cohort **(D–F)**.

**FIGURE 5:**
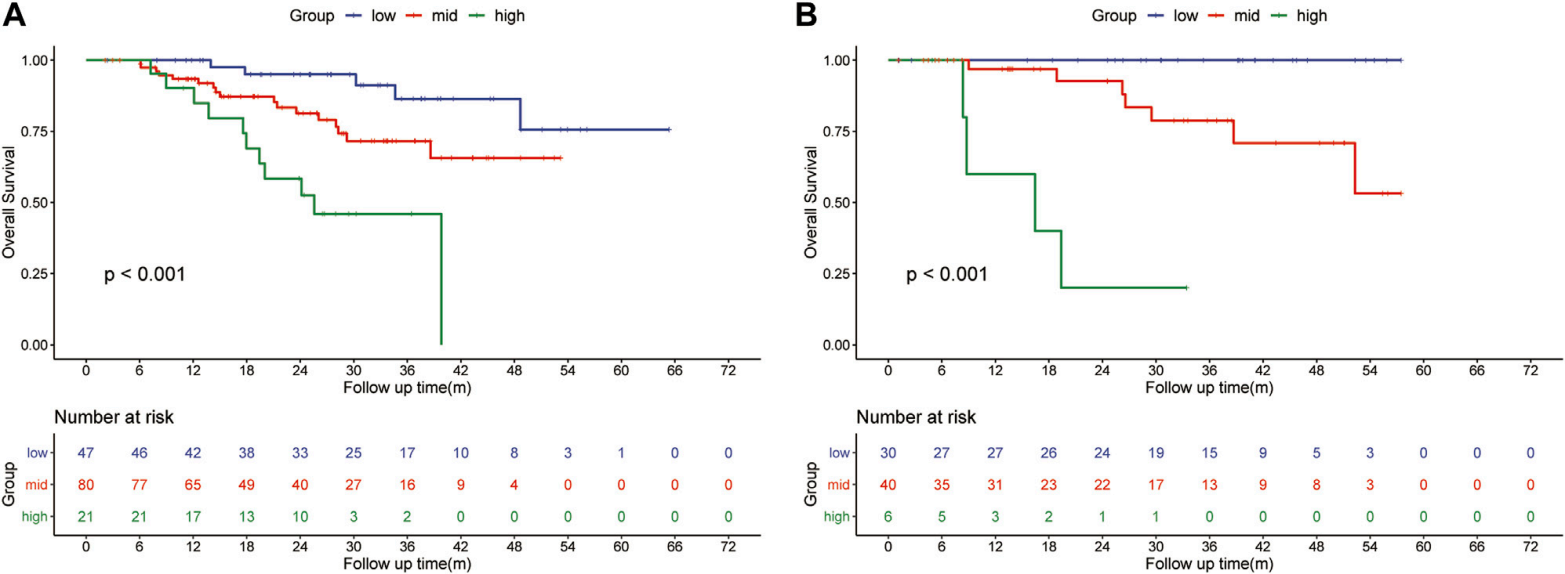
Kaplan-Meier survival curves for subgroups of patients estimating overall survival in the training cohort **(A)** and the internal validation cohort **(B)**.

The HLR group was randomly divided into two cohorts in the ratio of 1:2 as the training (*n* = 148) and validation cohorts (*n* = 76). Based on the independent risk factors identified in the multivariate analysis, preoperative tumor size, preoperative tumor number, PT, and response to HAIC were integrated to build a nomogram of OS (Figure 3C). Harrell’s C-indexes of OS prediction was 0.82 (95% CI 0.78–0.86) in the training cohort and 0.87 (95% CI 0.83–0.93) in the validation cohort. The calibration curves for the probability of 1-, 2- and 3-year OS also demonstrated good agreement between prediction by the nomogram and the actual observation in the training cohort (Figures 4A–C) and the validation cohort (Figures 4D–F). The discrimination power of the nomogram was analyzed by stratifying the predicted OS probabilities into three groups. Patients could be classified into low risk (score ≤ 104), middle risk (score = 104–135), and high risk (score >135) of mortality, and the three groups represented a distinct prognosis in the training (*p* < 0.001, Figure 5A) and validation cohorts (*p* < 0.001, Figure 5B).

## 4 Discussion

Preoperative treatments for HCC are lacking to date. Here in, we find that preoperative FOLFOX-HAIC prolongs OS and RFS in HCC patients who underwent liver resection. Furthermore, we have developed a nomogram that performs efficiently in predicting the OS of patients who underwent FOXFOX-HAIC followed by liver resection.

The ORR of FOLFOX-HAIC is found to be high (49.6%) in this study, which is similar to that (46.0%) reported by Li et al (2022). HAIC is a local therapy that directly delivers chemotherapeutics into tumor-associated arterial branches affording increased local drug concentrations. Because of a greater first-pass effect in the liver, HAIC provides stronger antitumor efficacy than systemic chemotherapy. Moreover, the pharmacokinetic profile of HAIC and the cytotoxic mechanism suggest that oxaliplatin-based HAIC may confer greater efficacy against HCC compared to cisplatin-based HAIC (Dzodic et al, 2004; Bruno et al, 2017).

Preoperative TACE for HCC was reported in several previous studies, but the benefit is controversial. Some studies reported that TACE before liver resection demonstrated a significant effect in improving surgical outcomes (Hsu et al, 1986; Yu et al, 1993). Reciprocally, the study by Lee et al (2009) indicated that TACE before liver resection is not only correlated with higher mortality rates but is also a waste of medical resources. Preoperative treatments are efficacious generally in patients with large tumor burdens. TACE is relatively inefficient in HCC patients with large tumor burdens exceeding the up-to-seven criteria (Kokudo et al, 2019). The study by Li et al (2022) confirmed that FOLFOX-HAIC exhibited superior efficacy over TACE for patients with large HCC, and 38 patients (24%) in the FOLFOX-HAIC group underwent curative surgical resection, compared to 18 patients (12%) in the TACE group. Furthermore, peritoneal adhesion is more severe in post-TACE-treatment patients than those after treatment with HAIC, which poses difficulty to surgeons during operation. Therefore, HAIC is more suitable as a preoperative treatment compared to TACE.

To identify the critical factors determining patient survival with preoperative FOLFOX-HAIC, we performed PSM individually according to initial or preoperative tumor characteristics. The results indicated that the HLR group was associated with longer survival in the initial PSM cohort rather than in the preoperative PSM cohort. If the tumor size and tumor number are reduced to the same in the LR group after preoperative FOLFOX-HAIC, the surgical results were improved to the same level compared to the LR group. Crucially, the result of multivariate Cox regression analysis revealed that the significant factors associated with OS were preoperative tumor size and number rather than initial tumor size and number.

The HLR group was found to have more patients with longer operation time and hepatic portal occlusion time compared with the LR group. However, the postoperative hospital stays were shorter in the HLR group. Furthermore, there were no significant differences observed between the two groups in terms of operative blood loss, total postoperative complications, and 90-day mortality. These findings may suggest that the post-HAIC surgical operation was more challenging for surgeons. However, it did not lead to worse surgical outcomes. Although total postoperative complications were not significantly different (*p* = 0.230, initial PSM cohort; *p* = 0.560, preoperative PSM cohort), the incidence rate of hepatic insufficiency (6.7% vs. 1.3%, initial PSM cohort; 7.0% vs. 0.9%, preoperative PSM cohort) and bile leakage (3.1% vs. 0.4%, initial PSM cohort; 3.3% vs. 0.5%, preoperative PSM cohort) was higher in the HLR group, which may be related to the deterioration in liver function caused by oxaliplatin-induced liver parenchymal injury (Rubbia-Brandt et al, 2004; Aloia et al, 2006).

To predict the OS of patients with preoperative FOLFOX-HAIC, we developed a novel nomogram model. In addition to preoperative tumor size and preoperative tumor number, the response to HAIC, PT, and tumor differentiation were the significant factors associated with OS. The factors that we can obtain before surgery including preoperative tumor size, preoperative tumor number, the response to HAIC, and PT were selected to construct the nomogram as we aim to establish a model that can predict the OS before surgery. Hence, tumor differentiation was excluded. The study by Lei et al (2016) reported that responders were associated with longer survival than non-responders who underwent TACE followed by HCC resection and they considered response to TACE as a selection criterion for HCC resection. Similar to Lei et al (2016)’s observations, we found that responders were associated with longer survival than non-responders who underwent HAIC followed by resection of HCC. The response to preoperative treatment may be a measure of tumor biology. Thus, the inclusion of the response to HAIC is essential to improve the predictive performance of the nomogram. In addition to oncological factors, liver function is also a critical factor impacting the long-term survival of HCC patients. PT serves as a measure of the liver’s synthetic function. In the context of patients undergoing liver resection, the Model for End-stage Liver Disease (MELD) score (Kamath et al, 2001), which includes PT-INR, serum bilirubin, and serum creatinine, has been utilized to predict postoperative mortality risk (Teh et al, 2005). Notably, PT-INR has been demonstrated to have the most substantial influence on the MELD score and is indicative of liver functional reserve (Porte et al, 2010).

Based on the nomogram scores, patients in the HLR group were stratified into three subgroups with distinct prognoses. As all factors in the nomogram can be obtained before surgery, it is helpful to guide surgeons to select the proper candidates to receive liver resection post FOXFOL-HAIC. FOLFOX-HAIC is performed every 3 weeks and efficacy is assessed every 6 weeks. Once every estimation is completed, the nomogram can be performed. If patients belong to the low-risk group, liver resection should be considered by the surgeons, while if they fall into the middle- or high-risk group, surgeons are suggested to select FOXFOL-HAIC treatment or other palliative alternatives.

Despite several merits, the current study has several limitations that should be noted. Firstly, the retrospective nature of this study may have introduced some degree of selection bias. Secondly, the results were based on a population of patients with HCC from an area with a predominant prevalence of HBV infection, and therefore the applicability of preoperative FOLFOX-HAIC for HCC patients with other underlying etiologies requires further investigation. Lastly, since this was a single-center study, external validation of the nomogram was not available.

In conclusion, preoperative FOLFOX-HAIC is associated with a longer survival outcome for HCC patients. The critical factors determining the survival of patients with preoperative FOLFOX-HAIC were preoperative tumor characteristics rather than initial tumor characteristics. Responders were associated with longer survival than non-responders who underwent HAIC followed by resection of HCC. Furthermore, the novel nomogram developed in this study could efficiently predict the OS of patients who underwent preoperative FOLFOX-HAIC.

## Data Availability

The raw data supporting the conclusion of this article will be made available by the authors, without undue reservation.

## Abbreviations

AFP, alpha-fetoprotein; ALBI, albumin-bilirubin; ALT, alanine aminotransferase; AST, aspartate aminotransferase; CR, complete response; FOLFOX, cisplatin, fluorouracil, and leucovorin; HAIC, hepatic arterial infusion chemotherapy; HBsAg, hepatitis B surface antigen; HBV, hepatitis B virus; HCC, hepatocellular carcinoma; mRECIST, modified Response Evaluation Criteria in Solid Tumors; MRI, magnetic resonance imaging; MVI, microvascular invasion; ORR, overall response rate; OS, overall survival; PD, progressive disease; PR, partial response; PSM, propensity score matching; PT, prothrombin time; PVTT, portal vein tumor thrombus; RFS, recurrence-free survival; SD, stable disease; TACE, transhepatic arterial chemotherapy and embolization.
